# Comparative genomic analysis of nine *Sphingobium* strains: insights into their evolution and hexachlorocyclohexane (HCH) degradation pathways

**DOI:** 10.1186/1471-2164-15-1014

**Published:** 2014-11-23

**Authors:** Helianthous Verma, Roshan Kumar, Phoebe Oldach, Naseer Sangwan, Jitendra P Khurana, Jack A Gilbert, Rup Lal

**Affiliations:** Molecular Biology Laboratory, Department of Zoology, University of Delhi, Room No. 115, Delhi, 110007 India; Interdisciplinary Centre for Plant Genomics & Department of Plant Molecular Biology, University of Delhi, South Campus, New Delhi, India; Argonne National Laboratory, 9700 South Cass Avenue, Argonne, IL 60439 USA; Department of Ecology and Evolution, University of Chicago, 5640 South Ellis Avenue, Chicago, IL 60637 USA

**Keywords:** Hexachlorocyclohexane (HCH), *Sphingobium*, *lin* genes, Xenobiotic compounds

## Abstract

**Background:**

*Sphingobium* spp. are efficient degraders of a wide range of chlorinated and aromatic hydrocarbons. In particular, strains which harbour the *lin* pathway genes mediating the degradation of hexachlorocyclohexane (HCH) isomers are of interest due to the widespread persistence of this contaminant. Here, we examined the evolution and diversification of the *lin* pathway under the selective pressure of HCH, by comparing the draft genomes of six newly-sequenced *Sphingobium* spp. (strains LL03, DS20, IP26, HDIPO4, P25 and RL3) isolated from HCH dumpsites, with three existing genomes (*S. indicum* B90A, *S. japonicum* UT26S and *Sphingobium* sp. SYK6).

**Results:**

Efficient HCH degraders phylogenetically clustered in a closely related group comprising of UT26S, B90A, HDIPO4 and IP26, where HDIPO4 and IP26 were classified as subspecies with ANI value >98%. Less than 10% of the total gene content was shared among all nine strains, but among the eight HCH-associated strains, that is all except SYK6, the shared gene content jumped to nearly 25%. Genes associated with nitrogen stress response and two-component systems were found to be enriched. The strains also housed many xenobiotic degradation pathways other than HCH, despite the absence of these xenobiotics from isolation sources. Additionally, these strains, although non-motile, but posses flagellar assembly genes. While strains HDIPO4 and IP26 contained the complete set of *lin* genes, DS20 was entirely devoid of *lin* genes (except *linKLMN*) whereas, LL03, P25 and RL3 were identified as *lin* deficient strains, as they housed incomplete *lin* pathways. Further, in HDIPO4, *linA* was found as a hybrid of two natural variants i.e., *linA1* and *linA2* known for their different enantioselectivity.

**Conclusion:**

The bacteria isolated from HCH dumpsites provide a natural testing ground to study variations in the *lin* system and their effects on degradation efficacy. Further, the diversity in the *lin* gene sequences and copy number, their arrangement with respect to IS*6100* and evidence for potential plasmid content elucidate possible evolutionary acquisition mechanisms for this pathway. This study further opens the horizon for selection of bacterial strains for inclusion in an HCH bioremediation consortium and suggests that HDIPO4, IP26 and B90A would be appropriate candidates for inclusion.

**Electronic supplementary material:**

The online version of this article (doi:10.1186/1471-2164-15-1014) contains supplementary material, which is available to authorized users.

## Background

The family *Sphingomonadaceae* has been subdivided into five genera: *Sphingomonas*, *Sphingobium*, *Novosphingobium*, *Sphingopyxis* and *Sphingosinicella*[1, 2]. To date, the genomes of nearly 40 sphingomonads have been sequenced, which has revealed the genetic basis for the degradation of a broad range of polycyclic aromatic hydrocarbons (PAH) and polysaccharides [3]. However, *Sphingobium* spp. are of particular interest due to their ability to degrade hexachlorocylcohexane (HCH). The majority of HCH isomers (i.e. α, β, δ, ϵ) are formed during the production of the insecticide lindane (γ-HCH), and have been active pollutants since the 1950s [4]. Among all these isomers, only γ-HCH has insecticidal properties. Purification of γ-HCH (10-12%) from the mixture leads to the formation of HCH muck (88-90% of the total HCH mixture) having mainly α (60-70%), β (5-12%), δ (6-10%), and ϵ (3-4%) isomers [5]. This has been generally discarded in the open by the side of industrial units creating a large number of HCH dumpsites between the 1960s to the 1980s around the world [6]. *Sphingobium* spp. are often enriched in HCH dumpsites and have been shown to acquire and maintain genes associated with HCH degradation [7–10].

**Figure 1:**
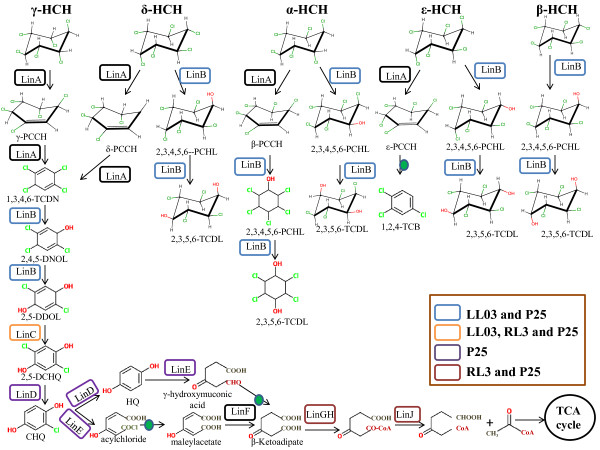
**Degradation of α-, β-, γ-, δ- and ϵ-HCH mediated by** ***lin*** **Pathway in** ***Sphingobium*** **spp.** Each color that encircles a specific *lin* gene depicts that the pathway is blocked within these *Sphingobium* strains*.* The compounds that are formed during HCH degradation are broadly known as PCCH: pentachlorocyclohexane, PCHL: pentachlorocyclohexanol, TCDN: tetrachloro-1,4-cyclohexadiene, DNOL: trichloro-2,5-cyclohexadiene-1-ol, DDOL: dichloro-2,5-cyclohexadiene-1,4-diol, TCDL: tetrachlorocyclohexanediol, DCHQ: dichlorohydroquinone, CHQ: chlorohydroquinone, HQ: hydroquinone. Green dots represent spontaneous reaction.

The degradation potential for HCH isomers has been attributed to the *lin* pathway (Figure 1), which has been studied in detail in both *Sphingobium japonicum* UT26S [11, 12] and *Sphingobium indicum* B90A [6]. The *lin* pathway is subdivided into an upper degradation pathway consisting of HCH dehydrochlorinase (LinA), haloalkane dehalogenase (LinB) and dehydrogenase (LinC/LinX), and a lower degradation pathway consisting of reductive dechlorinase (LinD), ring cleavage oxygenase (LinE), maleylacetate reductase (LinF), an acyl-CoA transferase (LinG, H), a thiolase (LinJ) and transcription factors (LinI and LinR). The LinK, LinL, LinM and LinN i.e., a permease, ATPase, periplasmic protein and a lipoprotein respectively, together constitute a putative ABC-type transporter [6].

There is evidence that indicates high levels of polymorphisms in the amino acid sequences of the *linA* and *linB* genes. Further studies have revealed that these differences contribute to the efficacy of HCH degradation and substrate specificity [13]. While there are several strains of sphingomonads isolated from HCH dumpsites with demonstrated differences in HCH degradation ability [8, 14], genome-wide comparative analyses to better understand the *lin* pathway, localization of *lin* genes in the genome and methods of recruitment have not yet been undertaken.

In order to understand the evolution of the HCH-degradation pathway, the draft genomes of six *Sphingobium* spp. isolated from HCH dumpsites and the complete genomes of three previously-sequenced, well-studied strains were analysed. Here, we characterize the genetic divergence between these strains in reference to the *lin* catabolic system and auxiliary characteristics associated with bioremediation potential. We also present evidence for possible plasmid and IS*6100* based horizontal gene transfer (HGT) as the method for spread of the *lin* system genes among sphingomonads. Additionally, variation in the *lin* gene sequences is a matter of further investigation for improved degradation ability of these strains.

## Results and discussion

### Genomic features of *Sphingobium*strains

**Figure 2:**
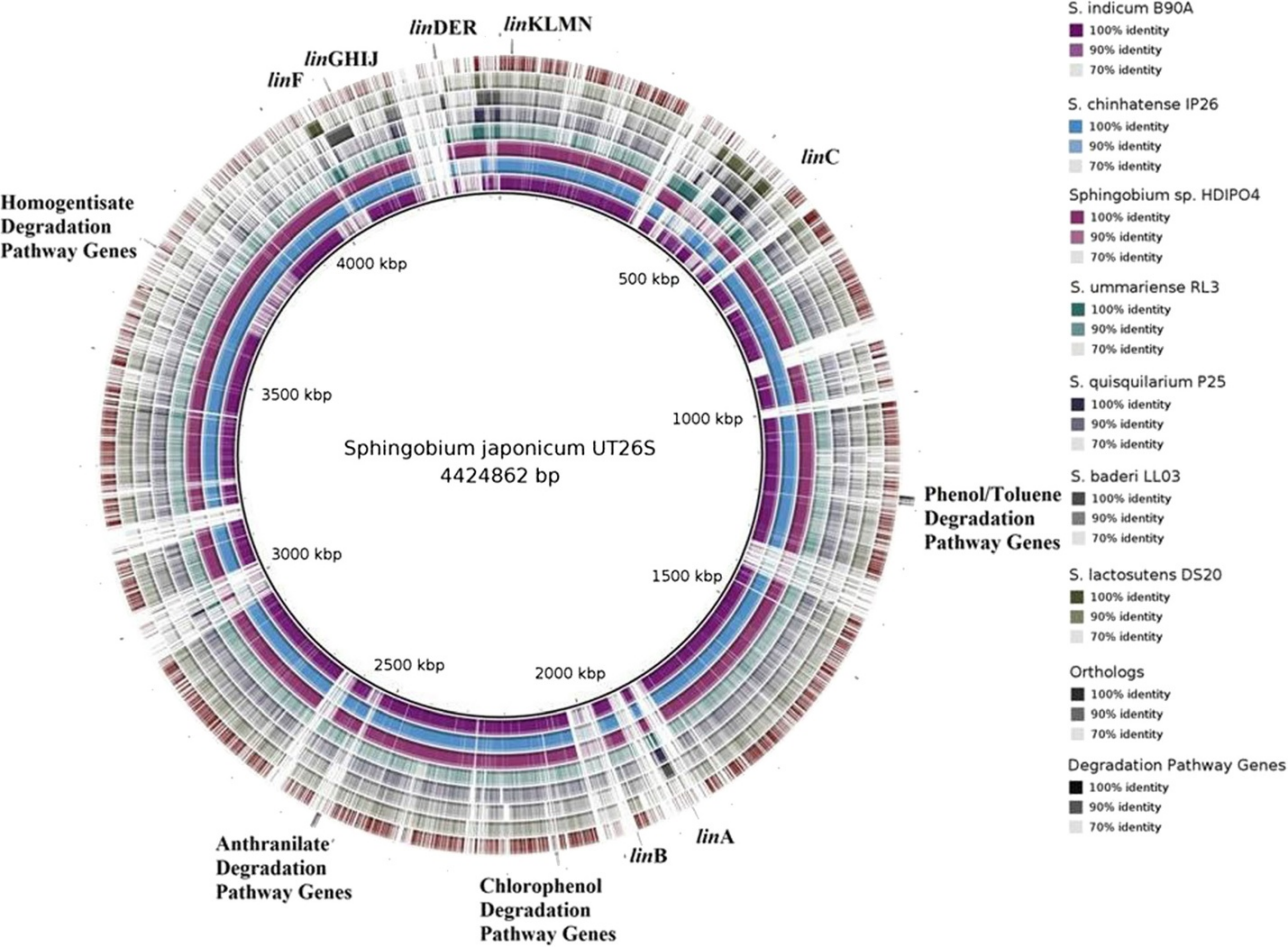
**Comparative genome map of** ***Sphingobium*** **spp.** Mapped (BLASTN) over *Sphingobium japonicum* UT26S as a reference genome in which all genetic elements of UT26S were concatenated in the order of Chr1 (3,514,822 bp), Chr2 (681,892 bp), pCHQ1 (190,974 bp), pUT1 (31,776 bp) and pUT2 (5398 bp). Genes for HCH, Phenol/Toluene, Chlorophenol, Anthranilate and Homogentisate degradation pathways are identified in the outermost region of the figure. Genetic breakpoints between UT26S and other *Sphingobium* spp.; from the outside in; outermost circle1: Orthologous genes, circle 2: Draft genome of *S. lactosutens* DS20, circle 3: Draft genome of *S. baderi* LL03, circle 4: Draft genome of *S. quisquilarium* P25, circle 5: Draft genome of *S. ummariense* RL3, circle 6: Draft genome of *Sphingobium sp.* HDIPO4, circle 7: Draft genome of *S. chinhatense* IP26, circle 8 (innermost circle): Draft genome of *S.indicum* B90A. (Higher color intensity represents higher percentage identity *i.e.,* darker shades show higher sequence identity).

The genome sizes for the six newly sequenced *Sphingobium* spp. averaged 4.83 Mbp and ranged from 4.08 to 5.89 Mbp, with *S. chinhatense* IP26 maintaining the largest genome (Table 1). These sizes are consistent with existing *Sphingobium* spp. [15]. The variation in genome size can be partially correlated to the presence of genomic islands; IP26 maintained the largest genome and the highest genomic island content, while LL03 had the least (Table 1). This potentially reflects differential degrees of HGT and mobile genetic element acquisition among these strains. UT26S, B90A, IP26 and HDIPO4 all shared high sequence identity (>97%), whereas LL03, P25, RL3 and DS20 have accumulated more sequence variation despite being under similar selection pressures (90-70%) (Figure 2).

**Table 1:** **General characteristic features of the** ***Sphingobium*** **genomes**

Strains	IP26	HDIPO4	RL3	P25	DS20*	LL03*	B90A	UT26S	SYK6
**Project ID**	**PRJNA208542**	**PRJNA201012**	**PRJNA208544**	**PRJNA201016**	**PRJNA201649**	**PRJNA202090**	**PRJNA50313**	**PRJDA19949**	**PRJNA73353**
**NCBI Accession No.**	**AUDA00000000**	**ATDO00000000**	**AUWY00000000**	**ATHO00000000**	**ATDP00000000**	**ATIB00000000**	**AJXQ00000000**	**AP010803 to AP010806**	**AP012222, AP012223**
**Source of isolation**	**HCH Dumpsite, India**	**HCH Dumpsite, India**	**HCH Dumpsite, India**	**HCH Dumpsite, India**	**HCH Dumpsite, India**	**HCH Dumpsite, Czech Republic**	**Rhizosphere Soil, India**	**Soil Contaminated with γ-HCH, Japan**	**Waste water of kraft mill pulp, Japan**
**HCH Degarder**	**Yes**	**Yes**	**Yes**	**No**	**No**	**No**	**Yes**	**Yes**	**No**
**Genome Size (bp)**	**5,894,129**	**4,741,576**	**4,754,053**	**4,170,546**	**5,360,246**	**4,848,216**	**4,082,196**	**4,424,878**	**4,348,133**
**G + C content (%)**	**64.5**	**65.5**	**65**	**64.7**	**63.6**	**64**	**65.8**	**65.6**	**66**
**Predicted CDS**	**4646 (5161068 bp)**	**4646 (4105641 bp)**	**4636 (4152861 bp)**	**4033 (3644184 bp)**	**5288 (4682346 bp)**	**4914 (4312911 bp)**	**3976 (3570642 bp)**	**4414 (3929727 bp)**	**4097 (3825558 bp)**
**Pseudogenes**	**10**	**46**	**2**	**70**	**45**	**39**	**-**	**-**	**-**
**Average Gene Size (bp)**	**903**	**889**	**896**	**903**	**890**	**852**	**886**	**890**	**933**
**% of CDS**	**87.56%**	**86.58%**	**87.35%**	**87.38%**	**88.31%**	**88.96%**	**87.46%**	**88.80%**	**87.98%**
**IS** ***6100***	**21**	**18**	**19**	**24**	**15**	**22**	**11**	**5**	**0**
**tRNA**	**66**	**54**	**56**	**49**	**59**	**56**	**54**	**55**	**50**
**rRNA**	**11**	**13**	**12**	**9**	**15**	**12**	**3**	**9**	**6**
**Genomic islands (bp)**	**1104688**	**705420**	**180116**	**201158**	**179141**	**48062**	**427792**	**395714**	**244403**

*Strain having CRISPR element.

CRISPR elements were only found associated with *S. baderi* LL03 (22 spacers) and *S. lactosutens* DS20 (5 spacers). These spacer sequences are known bacterial defense mechanisms against viral and plasmid challenges acquired from foreign invading DNA, with the number of new phage-derived spacers being correlated with phage resistance [16]. However, their spacer sequences had no similarity to known viral phage sequences. Furthermore, LL03 maintained a type II CRISPR element with the *cas9* gene involved in target interference, whereas DS20 had type I CRISPR elements with the *cas3* gene [17]. Strains LL03 and DS20 were isolated from HCH dumpsites in the Czech Republic and India, respectively, and these strains had two different CRISPR/CAS systems, that may correspond to their different geographical locations. These data also reflected that LL03 should have the greatest phage resistance.

### Comparative phylogenetic analysis

**Figure 3:**
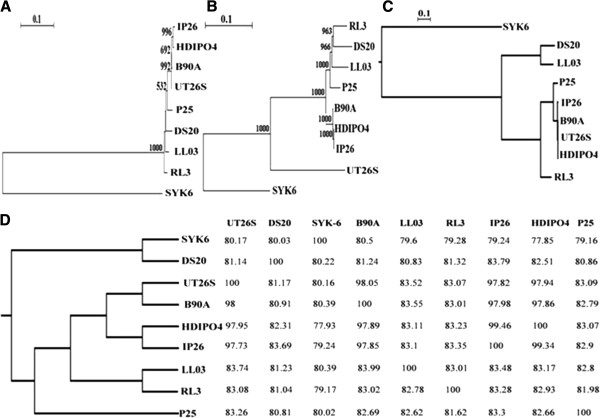
**Phylogenic constructions of** ***Sphingobium*** **spp.** *Sphingobium baderi* LL03, *S. lactosutens* DS20, *S. chinhatense* IP26, *S. quisquilarium* P25, *S. ummariense* RL3, *Sphingobium* sp. HDIPO4, *S. indicum* B90A, *S. japonicum* UT26S and *Sphingobium* sp. SYK6. **(A)** 16S rRNA gene sequence-based phylogeny was performed with the neighbor joining method using TreeconW 1.3b (bootstrap value = 1000) **(B)** The amino acid sequences of 28 single copy genes were concatenated for all ten genomes and phylogeny performed with the neighbor joining method using TreeconW 1.3b (bootstrap value =1000) **(C)** &**(D)** Whole genome based tetranucleotide correlation and ANI based pairwise comparisions between the genomes was carried out for all the genomes. A pearson correlation matrix was constructed on the basis of their tetranucleotide correlation and ANI values, followed by hierarchical clustering on the resultant matrix using MEV4.9.0, and visualized on MEGA4.

Four different phylogenetic methods (16S rRNA gene sequence, single copy gene sequences, tetranucleotide frequency based correlation, and average nucleotide identity (ANI)) were used to analyze the relationships of the nine strains under study (Figure 3). The consensus tree topology obtained by these methods clustered *S. indicum* B90A, *S. japonicum* UT26S, *S. chinhatense* IP26, and *Sphingobium* sp. HDIPO4, with the exception of the single copy gene approach. Notably, these four strains were the only ones with an entirely complete *lin* pathway, thus suggesting convergent evolution through HCH selection pressure. Furthermore, ANI topology supported the grouping of *Sphingobium* sp. HDIPO4 and *S. chinhatense* IP26 as subspecies (≥99.34%) (ANI values within the subspecies >98%) [18]. The other five strains i.e., LL03, DS20, RL3, P25 and SYK6 did not produce a consensus phylogeny, with relationships differing between these approaches; in short, strains with the complete *lin* pathway formed a closed group whereas, the others have diverged. In addition, 16S rRNA and single copy gene approaches may be problematic for differentiation among highly related strains (as these methodologies do not consider the influence of HGT). However, ANI based pairwise comparison has clustered LL03 and RL3 (partial *lin* gene deficient but HCH degraders) in a monophyletic clade with P25 (partial *lin* gene deficient and slow HCH degrader) forming a close relationship. Moreover, DS20 and SYK6 (non-HCH degraders) were clustered together. This suggests that ANI based phylogeny is more appropriate and mirrors their relationship with respect to HCH degradation.

### Common gene content and functional profiling of *Sphingobium*spp

**Figure 4:**
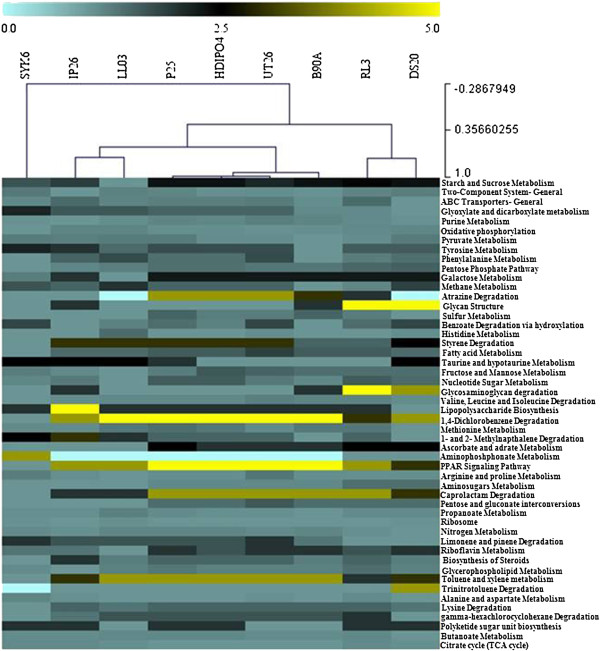
**Functional profiling of the** ***Sphingobium*** **genomes.** Heat map showing the normalized relative abundance of the top 50 subsystems enriched in the nine *Sphingobium* genomes. The strains and enriched pathways were clustered using Pearson correlation with a 0.8% minimum abundance. The color scale represents the relative abundance of gene content for each pathway, normalized by sample mean.

Core genome analysis identified 322 orthologs conserved between the nine genomes. The majority of these genes were involved in housekeeping functions such as the synthesis of ribosomal proteins, DNA replication, transcription & translation machinery, amino acid metabolism and membrane transporters. Core genome analysis for the eight strains that were either isolated from an HCH dumpsite or showed HCH degradation potential (i.e. all except SYK6) predicted 880 orthologs (Figure 2), which suggests a significant increase in genomic conservation (~2.7 times) resulting from the selective pressure of HCH exposure. This conservation is also seen in the degradation potential for other aromatic compounds such as benzoate, 1,4-dichlorobenzene, 1,2-methylnapthalene, caprolactam, toluene and xylene, trinitrotoluene, biphenyl and styrene degradation (Figure 4). Genes involved in the degradation of p-hydroxybenzoate, benzoate, quinate, gentisare, and catechol were also identified in the nine *Sphingobium* genomes (Additional file 1: Table S1). The presence of degradation pathways for phenol/toluene, chlorophenol, anthranilate, and homogentisate are identified in UT26S [19]. These pathways were observed in at least two of the newly sequenced strains (Additional file 1: Table S1). This suggests that these *Sphingobium* spp. possess broad aromatic compound degradation potential, although we did not observe the presence of these compounds at the HCH dumpsite [20]. The link between these aromatic degradation pathways and the HCH degradation pathway requires further investigation.

Functional profiling was used to analyze pathways that were differentially enriched in these strains. For this, a dendogram was constructed based upon the top 50 subsystems at 0.8% minimum abundance using pearson correlation distance. The analysis revealed that the two-component system for gene expression was highly abundant in all of the *Sphingobium* genomes (Figure 4). This system is known to facilitate adaptation to extreme environmental conditions and likely contributes to the ability to survive in conditions of high HCH pressure, salinity, and acidity that exist at the HCH dumpsite [9]. Additionally, the nine strains collectively showed an abundance of ABC transporters within their genomes. The abundance of these transporters implies that these strains are highly engaged in transport of a wide variety of substrates across extra- and intracellular membranes [21], which is consistent with the *Sphingobium* proficiency for degradation of a wide range of xenobiotics (mentioned above; Figure 4).

Interestingly, HDIPO4 and IP26, which had a close phylogenetic relationship, demonstrated differences in their functional repertoire, based on the top 50 subsystems. This is primarily driven by an increased abundance of 1,4-dichlorobenzene degradation, toluene and xylene degradation, caprolactam degradation, PPAR signaling and atrazine degradation pathways in HDIPO4, which was clustered with functional profiling of P25 and UT26S as they shared these enrichments. Furthermore, lipopolysaccharide biosynthesis, tyrosine metabolism, glycan and glycosaminoglycan degradation pathways were found enriched in IP26 as compared to HDIPO4. This variation suggests that while these strains exhibit similar genomic content, they exhibit differential dominance in their metabolic preferences.

### Nitrogen assimilation and the presence of flagellar genes in non-motile *Sphingobium*

The genomes of all the nine strains were found to contain an enrichment of the two component signal transduction system for nitrogen stress response (*NtrC* pathway) . Additionally, the large subunit of assimilatory nitrate reductase, a key regulator that potentially enables the utilization of nitrate as a nitrogen source, was found to be under diversifying natural selection (dN/dS = 1.09), which suggests that these strains can tolerate low inorganic nitrogen concentrations and are evolving in response to this inorganic nitrogen stress. At high nitrogen concentration, the transmembrane protein *glnC* (*ntrB*/Histidine kinase) responds to nitrogen availability and phosphorylates *glnG* (*ntrC*), which in turn leads to the activation of *glnA* (glutamine synthetase) [22]. Another key regulator of the pathway is *glnB*, which interacts and regulates the activity of *glnC*. When the nitrogen availability is low, *glnB* is subjected to post transcriptional modification by uridylation (mediated by *glnD*). This modification is reversed in N-sufficient conditions [22]. Thus, the presence of *NtrC* pathway and nitrate reductase genes explains the ongoing phenomena of nitrogen assimilation by these strains at HCH dumpsites to acclimatize themselves in such nitrate concentrations. Increasing exposure to elevated hydrocarbon concentrations was found to be positively correlated with the relative abundance of genes associated with nitrogen metabolism [23].

The *NtrC* pathway is also associated with genes regulating chemotactic response, such as *cheY*, *motA*, *motB*, and flagellar biosynthesis proteins, such as *flhA*, *fliO*, *fliP*, *fliR* etc. All these genes were also found in the core-genome. *cheY* modulates the cell’s ability to interact with the flagellum and controls swimming behavior [24]. Interestingly, while these *Sphingobium* strains are considered non-motile [25–30], each genome housed more than half of the genes needed for flagellar assembly and functioning. This raises the possibility that they are either in a process of acquiring or losing motility. The abundance of chemotaxis and motility genes has already been demonstrated in the metagenome of the HCH dumpsite [9] from where HDIPO4, IP26, P25, DS20, and RL3 were isolated. However, further analysis is needed to probe the reason for retention or loss of flagellar genes in the *Sphingobium* strains, and to investigate whether *Sphingobium* have the potential to gain motility through acquisition of the remaining genes under the high selective pressure of HCH in the stressed environments.

### Recruitment of *lin*pathway through different routes

The genome analysis revealed a mosaic distribution of *lin* genes and IS*6100* elements in HCH-degrading *Sphingobium* spp. coupled with high polymorphism levels in the *lin* genes. This indicates the recruitment of *lin* genes through different routes in *Sphingobium* spp. under HCH stress, and further that the pathway has not yet stabilized in these strains but is instead subjected to further rearrangements and polymorphisms.

### IS*6100*-mediated recruitment based on mosaic distribution pattern of *lin*genes

The IS*6100* elements, known for disseminating *lin* genes through HGT among sphingomonads [7, 31–33], were found to be present in all of the newly sequenced strains associated with HCH degradation, including strain DS20 which did not degrade HCH. The number, as determined from the genome sequence, varied from 5 copies in UT26S to 24 copies in P25 (Table 1). The presence of a large number of IS*6100* elements reflects a high degree of genomic rearrangement, as the IS*6100* elements have already been demonstrated to play an important role in the spread and reorganization of the *lin* pathway in sphingomonads [7, 10, 31–33].

To further explore the mechanism of HGT in the spread and diversification of the *lin* system, we examined the colocalization of *lin* genes with mobile elements such as the insertion sequence IS*6100* and transposons, and their presence on plasmids. In all of strains where *linA* gene was present in, it was found in nearly identical association with IS elements as in UT26S i.e. IS*6100* was found within proximity of <5Kbp. However, in RL3, two IS*6100* copies lies in the same orientation within the above mentioned range. Hence, this suggests that among these strains the association of *linA* with IS*6100* is consistent, but the reason and possible involvement of IS*6100* in the mechanism of duplication of the *linA* gene in RL3 needs to be identified.

**Figure 5:**
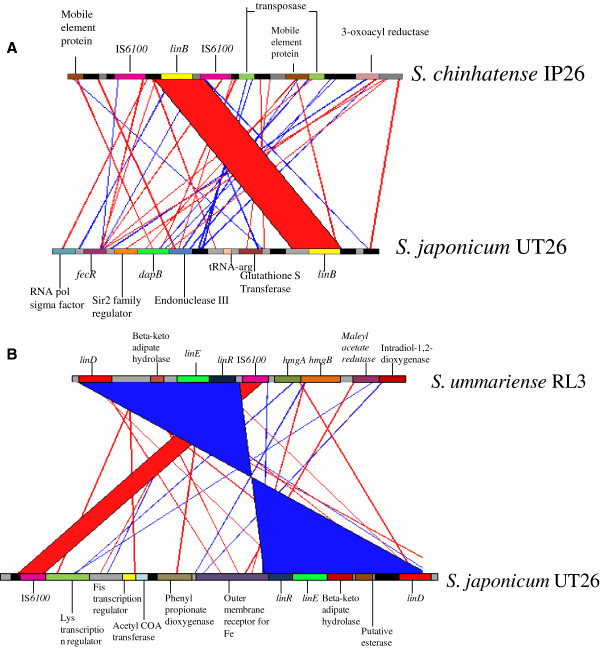
***lin*** **genes and their association with IS** ***6100*** **in HCH degrading** ***Sphingobium*** **spp. (A)** *linB* associated with IS*6100* at both its end in strain IP26 **(B)** association of *linDER* with homogentisate degradation pathway genes *hmgA* and *hmgB* separated by IS*6100* in strain RL3.

In IP26, *linB* was found to be flanked on both sides by IS*6100* (Figure 5A). Additionally, resolvase genes were found at the flanking ends of both of these transposons. Strains LL03 and P25 did not contain the *linB* gene, as confirmed by PCR amplication. Thus, either these strains have yet to acquire the *linB* gene, or, given the flanking IS*6100* elements, it is suggested that the loss of *linB* could have occurred via an intra-chromosomal single homologous recombination between two copies of IS*6100*[19, 10].

In HDIPO4, a truncated copy of *linF* along with complete set of *linC* and *linB* was found with an IS*6100* element (length of the segment = 15 Kbp) (Figure 5B). In contrast, in the case of the reference UT26S, these elements were dispersed, with *linF* present on chromosome 2 and *linB* and *linC* on chromosome 1. The association of these three elements suggests that they may have been brought together by IS*6100*-mediated transposition, a hypothesis supported by the fact that HDIPO4 contains a high number of IS*6100* comparable to UT26S (Table 1), and that they may be in the process of forming an operon.

Of the three copies of *linDER* present in RL3, one was closely associated with the *hmgB* and *hmgA* genes of the homogentisate degradation pathway, separated by a copy of IS*6100* (Figure 5B). In contrast, in UT26S, *linDER* was housed on a plasmid (pCHQ1), while *hmgB* and *hmgA* were found on chromosome 2 [19]. Therefore, in RL3, it is possible that these two different aromatic compound degradation pathways were brought into close proximity by IS*6100* mediated transposition. Thus, IS*6100*, apart from the spread of *lin* gene system, might be effective in the spread of homogentisate pathways despite the absence of homogentisate selective pressure at the HCH dumpsite, consistent with the fact that already sphingomonads that degrade aromatic hydrocarbons were found to contain catabolic genes associated with IS*6100*[34].

In strain LL03, isolated from the Czech Republic, *linGHIJ* genes were associated with IS*6100*, whereas in the UT26S genome, isolated from Japan, IS*6100* was absent from the region proximal to these lower pathway genes. As IS*6100* is reported to be a key driver in the recruitement of the *lin* system [6], differential organization of the IS*6100* element with respect to *lin* genes for strains from geographically-disparate locations reflects an ongoing IS*6100*–driven evolution of the *lin* system, including the lower degradation pathway components such as *linGHIJ*.

IS*6100* elements have also been found in the genome of DS20, which did not degrade HCH isomers (due to the lack of *lin* genes except *linKLMN*). However, in DS20, the regions flanking the IS*6100* elements comprised a variety of xenobiotic tolerance and degradation genes (i.e., benzene 1,2-dioxygenase, CopA family copper resistance protein, maleylacetatereductase, a putative efflux protein, chlorocatechol 1,2-dioxygenase), which further supports the role of IS*6100* in distributing genes for a broad-range of such functions in *Sphingobium* spp. The fact that DS20, a non-HCH degrader, maintained 15 copies of IS*6100* elements clearly suggests the potential of this strain to acquire *lin* genes through IS*6100* mechanisms in the future.

### Plasmid mediated recruitment

In investigating the presence and spread of the *lin* genes, the recently sequenced genome of an HCH-degrader *Sphingomonas* sp. MM-1 is of interest as it was found to have five plasmids housing the genes of the *lin* pathway [35]. In the MM-1 genome, the *linF* was found on pISP0; *linA, linC,* and a truncated *linF* on pISP1, *linDER* on pISP3, and *linB, linC,* and another truncated *linF* on pISP4 [35] and *linGHIJ* was found on pISP0. Genes for an ABC transporter were found on the chromosome, but these did not share at least 80% identity to the *linKLMN* genes of UT26S. In addition to this, in strain UT26S, HCH-specific genes of the *lin* pathway were found to be housed on regions unique to the UT26S genome [19]; with *linA, linB, linC* genes in chromosome 1, *linF* on chromosome 2, and *linDER* on the plasmid pCHQ1 [19]. The lower pathway genes, including *linGHIJ* and *linKLMN* were found on chromosomes 2 and 1, respectively, in regions that were conserved among sphingomonads [19].

**Figure 6:**
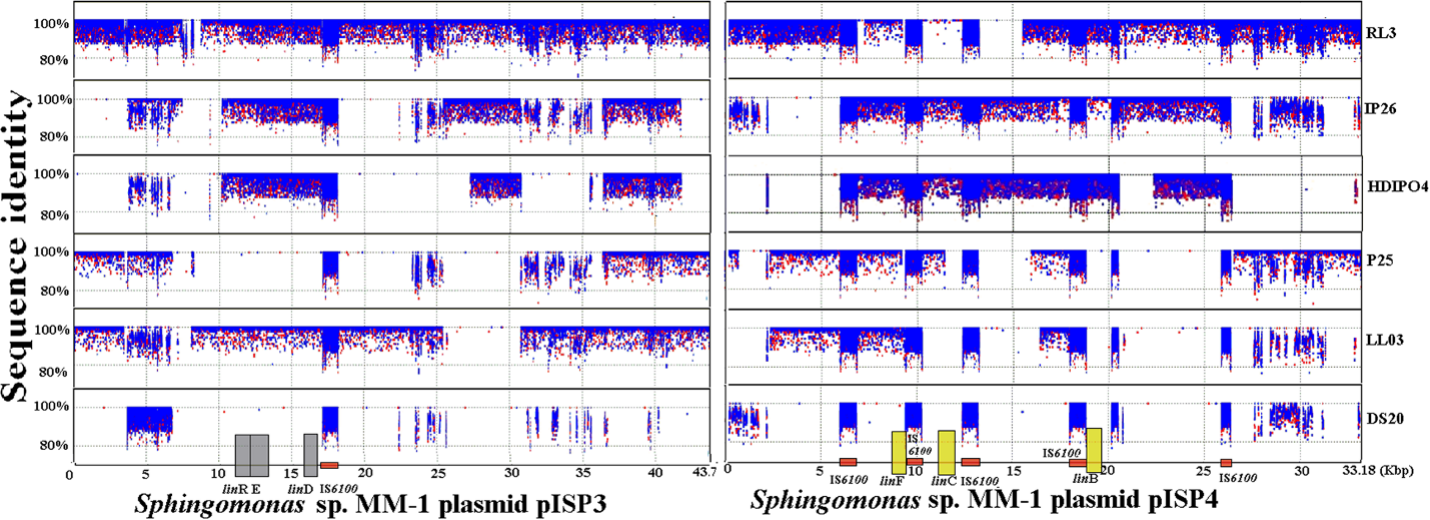
**Genome recruitment plot mapping on** ***Sphingomonas*** **sp MM-1 plasmid A) pISP3 and B) pISP4: the raw reads of the six novely sequenced strains were mapped on plasmids of** ***Sphingomonas*** **sp. MM1.** The orange bars are depicting IS*6100* while the lin genes were marked with grey and yellow bars showing their position in reference.

Genome recruitment plots were created to map the raw reads of the six novel-sequenced strains to *Sphingobium* plasmid sequences to investigate the possibility of these plasmids playing a role in transfer of the *lin* genes. MM-1 plasmids pISP3 and pISP4 in particular were found to have a high percentage of coverage which was maximum with *S. ummariense* RL3 (Figure 6). As pISP3 houses *linDER*, it is highly probable that plasmid uptake and duplication may explain the triplication of *linDER* in RL3. The recent metagenomics analysis of the HCH dumpsite also reflected the enrichment of pISP3, suggesting its availability for other sphingomonads strains present at the HCH dumpsite [10]. Furthermore, pISP4 encodes *linB, linC,* and *linF,* and similarly shows a high degree of coverage by RL3. Consistent with the absence of *linC* from the RL3 draft genome, which was confirmed by PCR amplification by using the primer 5′-GCGGATCCGCATGTCTGATTTGAGCGGC-3′ and 3′-GCCTCGAGTCAGATCGCGGTAAAGCCGCCGTC-5′, there is a gap in the coverage seen in the plasmid region containing *linC* (11,370 to 12,122 bp), which is a region flanked by two IS*6100* elements in MM-1 (Figure 6). This points to the possibility that the plasmid has undergone either acquisition in MM-1 or looping out from RL3 of the *linC* gene during the course of evolution, mediated directly by IS*6100*. Mapping the raw reads of the six newly-sequenced *Sphingobium* strains to the plasmid sequences for MM-1 and UT26S, several of the MM-1 plasmids, but none of the UT26S plasmids demonstrated a high degree of coverage. Additionally, the proportionally higher presence of *lin* genes on plasmids in MM-1 than in UT26S suggests that strain MM-1 acts as a reservoir for plasmids allowing for the effective spread of the *lin* system, and thus may be an important strain to include in the consortium development as a potential disseminator of the *lin* system. Also, strains sharing similar arrangement profile of *lin* genes with MM-1 i.e., RL3, IP26 and HDIPO4, should be included into designing a consortium.

### Strains in transition to acquire *lin*pathway

Of the nine sphingomonads under study, seven possessed components of the upper HCH degradation pathway to varying degrees of completion, and two, SYK6 and DS20, were completely devoid of them (Additional file 1: Table S2). SYK6 did not contain any components of the *lin* system and the DS20 genome contained only genes of the lower *lin* pathway- *linKLMN* an ABC transporter. Of the HCH-degraders, not every strain was found to house the complete array of *lin* genes characterized in UT26S or B90A. For instance, the P25 genome lacked *linB, linC, linDER, linGH, linI* and *linJ* genes while, strains RL3 and LL03 both lacked *linC* and LL03 lacked *linB,* as confirmed by PCR amplificiation (Additional file 1: Table S2). The differential composition of the *lin* system between these strains may be indicative of different steps in the evolution of the *lin* pathway, with IP26 in the stage of probable homologous recombination and looping out of *linB*, while LL03 shows potential gain of *linGHIJ* through IS*6100-*mediated HGT. Strain DS20 possesses ABC transporters and shows potential for acquisition of the *lin* genes, as it holds 15 copies of IS*6100,* while P25, in addition to the ABC transporter, has *linA* and *linF* but is yet to acquire the other *lin* genes.

### *lin*system sequence diversity and its effect on metabolic efficiency

#### Upper *lin*pathway

The upper pathway genes *linA*, *linB* and *linC* degrade γ-HCH and α-HCH, and additionally *linB* acts on β-HCH, leading to the formation of β-2,3,4,5,6-pentachlorocyclohexanol (β-PCHL) (Figure 1). As α- and β-HCH form the major components of contamination at the HCH dumpsite (>80%), both *linA* and *linB* are extremely important enzymes encoding HCH dehydrochlorinase and haloalkane dehalogenase, respectively (Figure 1). To gain deeper insights into the *lin* gene sequence diversity and its impact on HCH degradation, the genetic divergence of the *lin* system components was analyzed with respect to the copy number and nucleotide sequence divergence of the *lin* genes in both upper and lower degradation pathways, using B90A as a reference.

**Figure 7:**
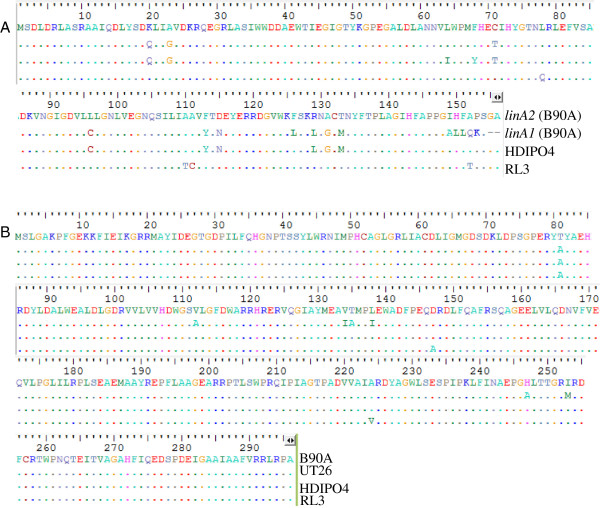
**A) Multiple sequence alignments of** ***linA*** **B)** ***linB*** **gene sequences in the strains showing variation.** Amino acid sequences of *linA* and *linB* genes from the strains were aligned using BioEdit to depict the substitutions at the respective sites.

The highest level of divergence in the upper HCH degradation pathway *lin* genes was reported for the *linA* gene encoding for HCH dehydrochlorinase in B90A [36]. The two previously characterized *linA* variants observed in B90A differed by 10% of their amino acid sequence, and were named *linA1* and *linA2.* The functional aspects of these variants have been well characterized, as they show enantioselectivity in (±) α-HCH degradation, with LinA1 selective for the (+) and LinA2 for the (-) α HCH [37]. Also, the degradation ability of LinA1 was found to be lower than that of LinA2 [36]. Among all the *lin* genes of the upper pathway, *linA* in the present study was found to be most diverged in HDIPO4, in which it appeared to be a hybrid of the two variants (*linA1* and *linA2*) with 94.8% sequence similarity to *linA1* and 92.9 to *linA2*. Near to the catalytic dyad D25 and H73 critical for its enzymatic activity, the HDIPO4 *linA* was found to be identical with *linA1*[38]. However, the C-terminal region corresponded to *linA2* (Figure 7A). This hybrid copy, now marked as *linA3,* requires further experimentation, but might be responsible for the comparatively better dehydrochlorination activity of HDIPO4 against α- and γ-HCH, as reported earlier [14].

Apart from this divergence of the *linA* sequence in HDIPO4, not such changes to the *linA* gene sequences were observed; all strains showed 100% sequence similarity to that of the *linA2* gene [36] with the exception that *linA2* of RL3 showed a single substitution of L78Q*.* It is important to mention here that the *linA* gene has already been reported to be under continuous selection pressure and a large number of variants of this gene exist [7, 32, 13, 39] and better variants of *linA* may be used for developing enzymatic bioremediation system for HCH.

In contrast to *linA,* there were less variations in *linB* sequences among strains under study. The sequence differences among *linA* and *linB* genes among different *Sphingobium* spp. are particularly interesting in light of findings that marginal differences in the amino acid sequences of *linB* in UT26S [40], SP^+^[41], B90A [31], BHC-A [42] and M1205 [43] can alter the efficacy and substrate range, with the former group degrading β-HCH to β-PCHL and the latter group taking the pathway beyond PCHL to TCHL. HDIPO4 housed two identical *linB* copies with a T81A substitution and overall 99.6% similarity to B90A while RL3 *linB* gene had 98.9% identity, with three substitutions (T81A, D147A and A224V) as compared to *linB* of B90A (Figure 7B). Here, the copy number difference is suspected to have a more impact, as the two copies of *linB* might explain the high β-HCH degradation efficacy of HDIPO4 [14, 27]. Apart from these two strains, no such diversity was observed, thus demonstrating the stability of *linB* gene in the population. *linC*, which encodes for HCH dehydrogenase was most conserved among the genes of the upper degradation pathway and demonstrated only a single substitution: Y172C in case of IP26. In any case these studies reflect that *linA* genes are more prone to evolutionary changes under HCH stress and have not stablized yet.

### Lower *lin*pathway

The lower pathway of γ-HCH degradation begins from 2,5-Dichlorohydroquinone (2,5-DCHQ) an intermediate of γ-HCH (Figure 1), which is mineralized by the lower pathway *lin* genes *(linDER, linF, linGHIJ,* and *linKLMN*) [6]. In contrast to the upper degradation pathway, very less is known about the divergence and polymorphisms of the genes of the lower degradation pathway.

**Figure 8:**
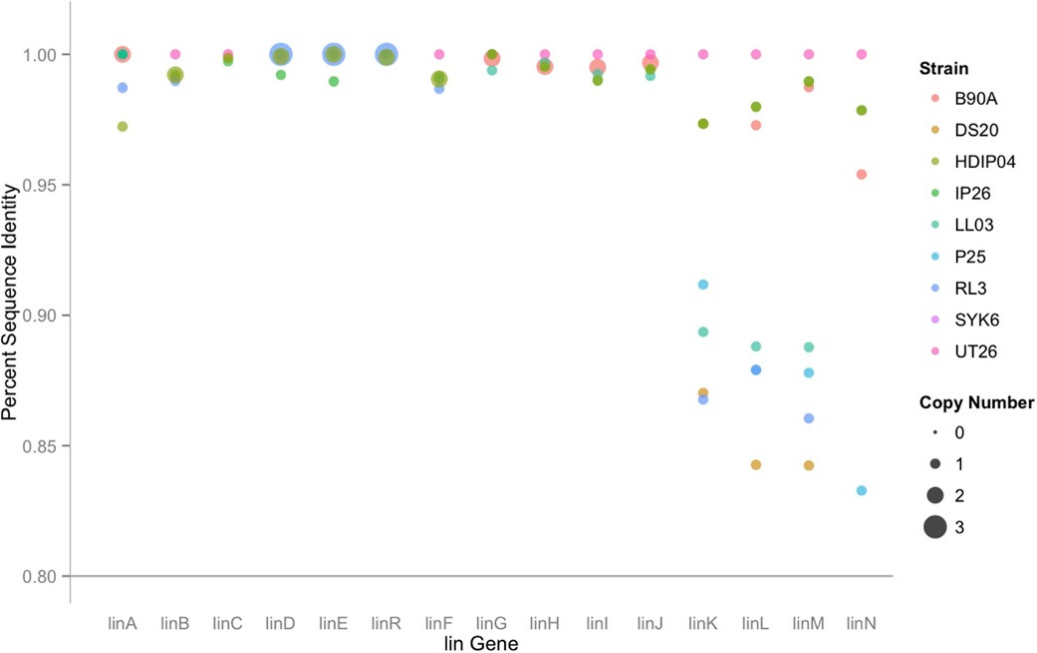
**Genetic sequence and copy number variation of** ***lin*** **genes: The genetic divergence, as quantified by percentage nucleotide identity to the archetypal strain UT26, and copy number variation in** ***lin*** **genes across the nine** ***Sphingobium*** **strains under study.**

Among all the *lin* genes of the lower pathway, *linF* was the most highly conserved, as its amino acid sequence was 100% identical in all genomes (Figure 8). In the *linDER* operon, the set of *linD* genes similarly showed minimal divergence, with the IP26, RL3, and LL03 genes sharing a substitution of N82S, and additionally IP26 having a substitution Q30P. Further, *linR* and *linE* had very little divergence; *linR* diverged only in one substitution in HDIPO4 (L12P) and *linE* was 100% identical in all strains. This highlights the fact that the *linDER* operon, which makes up the backbone of the downstream HCH degradation pathway, remained highly stable during the course of evolution. A greater degree of the variation of this operon was found, however, in copy number, as RL3 and HDIPO4 housed three and two copies, respectively (Figure 8 & Additional file 1: Table S2).

In particular *linGH, linI* and *linJ*, which mediate the later stages of the lower degradation pathway, i.e., conversion of β-ketoadipate to succinyl CoA and acetyl CoA (Figure 1), showed variation in the sequences of *linH* and *linI,* whereas *linG* and *linJ* sequences were 100% conserved among all these strains. Here, *linH* of HDIPO4 and IP26 were similar to each other, and both diverged from B90A with 99.06% identity. They held two substitutions (I31V and N171H) while LL03 shared the I31V substitution and additionally had a N131D substitution. *linI* was found to be identical in HDIPO4 and IP26, with a single substitution (A188T) and 99.62% identity to B90A, while LL03 had two substitutions (A9T and A185V) and 99.25% identity. However, the significance of sequence divergence in *linG, linH, linI,* and *linJ* genes among these strains is yet to be investigated.

Another important *lin* gene system of the lower pathway is the ABC transporter system i.e., *linK, linL, linM,* and *linN,* which encode a permease, ATPase, periplasmic protein, and lipoprotein, respectively. This ABC transpoter system is very important as it allows for the transport of HCH isomers and clearance of dead-end metabolites of HCH from the cell [21]. Out of the entire *lin* system these genes have shown the highest level of of divergence with *linK* at 86.8% in RL3, *linL* at 84.3% in DS20, *linM* at 84.2% in DS20 and *linN* at 83.3% in P25. Based on the prevalence of similar but non *lin*-specific ABC type transporters which are found by sequence identity searches across a variety of microbial species, it is hypothesized that the *linKLMN* operon derived from convergent evolution in response to environment changes. With the introduction of the HCH to the environment, pre-existing ABC-type transporters were likely recruited to the HCH degradation pathway, and thus several genetic variants might have undergone convergent evolution to select for transporters with increased efficiency for HCH-metabolite efflux, and these later generation genes were the one that subsequently underwent HGT. This is in contrast to the likely origin of the *linDER* operon, which encodes more highly HCH-specific enzymatic functions and is almost perfectly conserved, and thus was likely generated once, and spread through HGT from a single genetic ancestor. The *lin* pathway shows a characteristic pattern in which the upper HCH degradation pathway was diverged along a gradient from the most, *linA* (highly diverged) to the least, *linC* (least diverged). While in the lower degradation pathway *linKLMN* was the most highly diverged, followed by *linGHIJ*, *linDER* and *linF* respectively.

**Figure 9:**
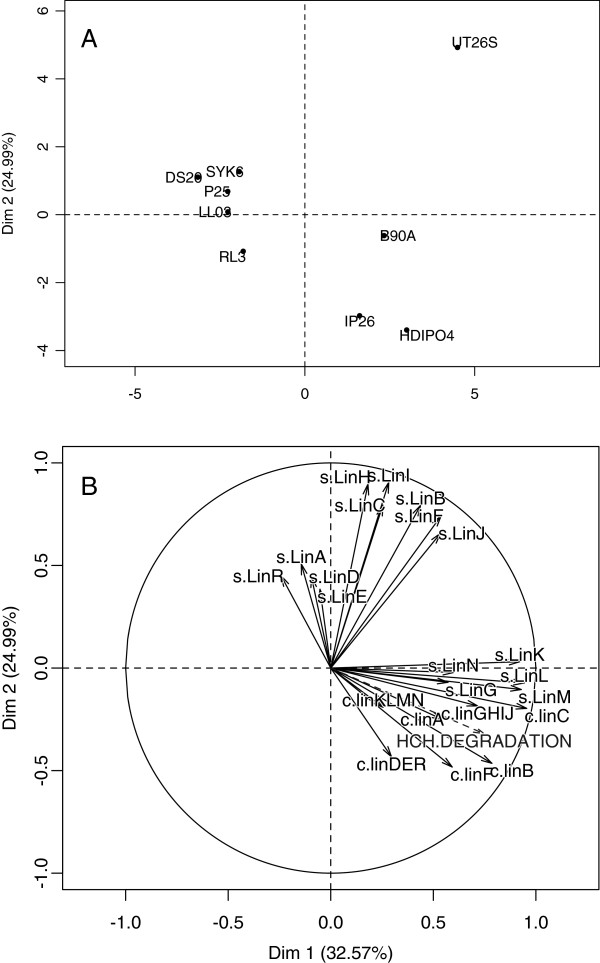
**Principal Component Analysis of genes involved in HCH degradation pathway. (A)** PCA individual factor plot showing the grouping of the nine *Sphingobium* strains based upon the sequence divergence and copy number of the set of *lin* genes. Principle components 1 (accounting for 30.87% of the variation of the strains) and 2 (14.47%) were chosen as the separation of the strains by these PCs demonstrated the highest fidelity to known HCH degradation ability. **(B)** PCA variable factor plot using principle component 1 and 2, showing the contribution of the lin genetic sequences (s.linA, s.linB…) and copy number (c.linA, c.linB…) to the variation of the nine *Sphingobium* strains. HCH degradation ability was plotted as a supplementary categorical variable (not factored into the PCs), with non-HCH degraders coded as 0, partial degraders as 1, and complete degraders as 2.

In literature, the degradation ability of these strains under consideration is in the order: HDIPO4>IP26>RL3. In order to further substantiate the relationship between *lin* system diversity and degradation efficiency, principle component analysis (PCA) was performed on the copy number and sequence divergence for these strains, with HCH degradation plotted as a supplementary variable to visualize correlation. The analysis demonstrated a close grouping of the four *lin*-deficient strains, i.e. SYK6, DS20, LL03, and P25 under PCA 1 and 2 (accounting for 32.57 and 24.99% of the variation in these strains, respectively) (Figure 9A). HDIPO4 and IP26 were colocalized in quadrant 4, opposite from the non-degrader cluster in terms of both dimension 1 and dimension 2, which is appropriate given that these two strains have degradation rates documented to be faster than archetypal strains UT26S and B90A [14] (Figure 9A). While copy number variation for *linF*, *linDER*, and *linB* were mapped most closely to the HCH degradation vector (Figure 9B), the copy numbers for *linC, linGHIJ* and again *linB* played a more important role in differentiating these strains, as they correlated significantly to PCA1 (p values 5.897964e-05, 2.998361e-02, and 1.206315e-02, respectively). In terms of sequence, *linA* and *linR* showed high correlation to degradation ability (Figure 9B), while *linL, linM, and linK* were significantly correlated to PCA1 (p values 1.340825e-04, 2.934465e-04, 4.192410e-04, respectively) and *linI, linH, linC, linB,* and *linF* were the most significant contributors to PCA2 (p values 0.0007954298, 0.0010696278, 0.0105835905, 0.0107931486, 0.0222238515, respecitvely). This suggests that while variation can be seen throughout the *lin* pathway and the copy number of *linF, linDER* and *linB* sequence for *linA* and *R* might have the most impact in optimizing the efficacy of an HCH enzymatic bioremediation system.

### Genes under diversifying natural selection

**Figure 10:**
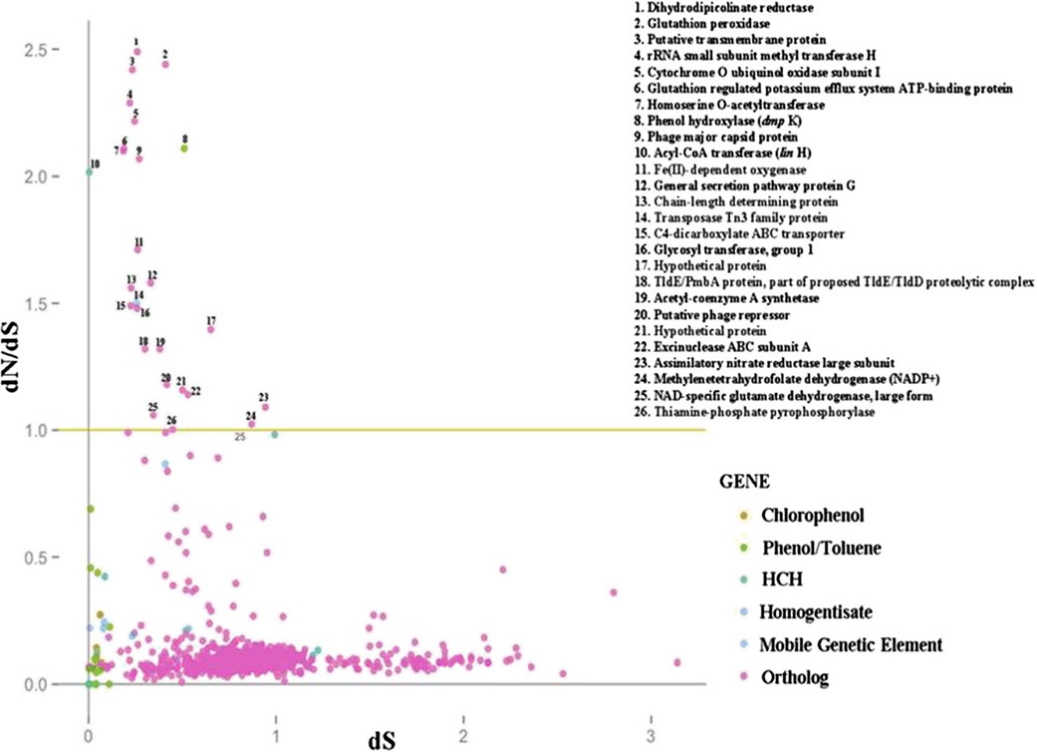
**dN/dS v/s dS plot of orthologs, xenobiotic degradation pathway genes and IS elements/integrases.** Sequences of orthologs, xenobiotic degradation pathway genes and IS elements/Integrases were used for calculating their dN/dS. Genes having dN/dS value >1 are under positive natural selection.

To identify the substitutions that have fixed along the independent lineages and their direction of evolution, dN/dS (rate of non-synonymous over synonymous substitution) analysis was performed for sets of orthologous genes, and in particular those responsible for the degradation of HCH, phenol/toluene, homogentisate, chlorophenol and transposons/integrases (Figure 10). The genes of interest found to be under diversifying selection (dN/dS > 1) includes those for Fe(II) dependent oxygenase, ABC transporters, assimilatory nitrate reductase, and general secretory pathway protein. These genes are largely associated with stress tolerance. As mentioned earlier, ABC transporters are involved in uptake of high molecular weight pharmacological agents including xenobiotic compounds [44]. Hence, these transporters and their activity are crucial for their capability of HCH degradation and other aromatic compounds and likewise, their positive selection indicates the importance of their function in the HCH-stressed environments from which these bacterial strains were isolated. Additionally, nitrate reductase catalyzes the conversion of nitrate into free nitrogen and likely would enable the *Sphingobium* spp. to more effectively enact a nitrogen stress response. Finally, the diversification of genes such as oxygenases, secretory pathway proteins and translocase components adds to the sphingomonads skill of degradation of a wide range of aromatic compounds. Notably, only *linH* has shown the dN/dS > 1, while the other *lin* genes did not. This clearly indicates that under strong HCH pressure, the whole *lin* pathway is likely to get stabilized in the population with more synonomous substitutions compared to non-synonomous ones and tends to be retained in the population.

## Conclusions

In sequencing the genomes of six novel *Sphingobium* species and comparing these to the known genomes of three other *Sphingobium* species, this study has begun to probe the natural variation in the *lin* pathway for HCH degradation. Analysis of the variation in the *lin* system, as well as in the phylogenetic relationships, core genomes, and functional profiles of these bacterial strains demonstrated unique characteristics of B90A, HDIPO4 and IP26 which could explain their higher efficacy as the degraders of HCH isomers. The information thus obtained can now be used to select these better-performing strains for the development of a bacterial consortium for on-site bioremediation of the HCH dumpsites. Focusing on the *lin* system, analysis of the similarities in the *lin* genes sequences and varying copy numbers between these strains has identified variations in the specific genes as key differentiators and these key components will be of critical interest as the most effective targets for optimization of an enzymatic bioremediation system. The analysis so far made reflect that better *linA* and *linB* variants can eventually be the ideal candidates for developing an enzymatic bioremediation system. Moreover, this study has uncovered evidence for genus-level HGT of plasmids housing components of the *lin* system, specifically between *Sphingomonas* sp. MM-1 and RL3. The additional *lin*-deficient strains are of further importance as they demonstrate varying degrees of acquisition of the *lin* system and will be useful in future homologous recombination studies to work with manipulated pathway completion through introduction of synthetic *lin* genes.

## Methods

### Selection and sequencing of the *Sphingobium*genomes

Six *Sphingobium* strains isolated from HCH dumpsites and demonstrating a range of HCH degradation abilities were selected for genome sequencing. Five of these *Sphingobium* strains i.e. *S. lactosutens* DS20^T^[26], *S. chinhatense* IP26^T^[27], *S. ummariense* RL3^T^[28], *S. quisquilarium* P25^T^[29], and *Sphingobium* sp. HDIPO4 were isolated from an HCH dumpsite in Chinhat village, Lucknow, India whereas, *Sphingobium baderi* LL03^T^[30], was isolated from an HCH dumpsite in Spolana, Czech Republic. In addition to these six strains, the genomes of an additional three strains: *Sphingobium indicum* B90A [45], *Sphingobium japonicum* UT26S [46], and *Sphingobium sp* SYK6 [47], were included as references in the study.

Genomic DNA was extracted from 5 ml pure culture pellets grown in Luria Bertani at 28°C until O.D. 1.0 or 1.2 using the SuperCos method [48]. DNA concentrations were quantified using NanoDrop spectrophotometer (NanoDrop Technologies Inc, Wilmington, DE, USA). For all the genomes, sequencing was performed using both the Illumina HighSeq 2000 and 454 GS-FLX Titanium platforms. For sequencing, a 2 Kbp paired end sequencing library was constructed, yielding ~100× coverage for each genome. An additional three *Sphingobium* genomes i.e. *Sphingobium japonicum* UT26S, *S. indicum* B90A and *Sphingobium* sp. SYK6 were retrieved to be used as references for this comparative analysis (Table 1).

### Genome assembly, annotation, and functional profiling

The sequencing data were assembled using ABySS 1.3.3 [49] at various k-mer lengths optimized for each genome. Detailed statistics of the genome assemblies are provided in Table 2. The assembly was validated using paired-end information on the Burrows-Wheeler Aligner 0.5.9 (BWA) [50]. The accession numbers and details of the genomes used in this study are provided under Table 1[51–56]. CDS were predicted using Glimmer-3 [57] and annotated on RAST 4.0 Server [58] for both the draft and complete genomes.

**Table 2:** **Detail statistics of genome assembly of** ***Sphingobium*** **spp.**

Organism (Genome status)	Assembler	K-mer length	No. of Contigs/Chromosomes & Plasmids	N50 (in Kb)
***S. baderi*** **LL03** ^**T**^ **(Draft)**	ABySS 1.3.3	47	92	269
***S. lactosutens*** **DS20** ^**T**^ **(Draft)**	ABySS 1.3.3	53	110	303
***Sphingobium*** **sp. HDIPO4 (Draft)**	ABySS 1.3.3	53	143	172
***S. chinhatense*** **IP26** ^**T**^ **(Draft)**	ABySS 1.3.3	61	236	142
***S. quisquilarium*** **P25** ^**T**^ **(Draft)**	ABySS 1.3.3	47	181	45
***S. ummariense*** **RL3** ^**T**^ **(Draft)**	ABySS 1.3.3	57	139	363
***S. indicum*** **B90A** ^**T**^ **(Draft)**	ABySS 1.2.7	41	149	54.5
***S. japonicum*** **UT26S** ^**T**^ **(Draft)**	PHRAP and CONSED	-	Chromosome 1 (3,514,822 bp), chromosome 2 (681,892 bp), pCHQ1 (190,974 bp), pUT1 (31,776 bp) ans pUT2 (5,398 bp)	-
***Sphingobium*** **sp. SYK6**	PHRED/PHRAP/CONSED	-	chromosome 1 (4,199,332 bp) and pSLGP (148,801 bp)	-

For functional profiling, coding sequences were extracted from the RAST server for all the genomes, and orthologous genes were determined using all-versus-all BLASTP at default parameters [59]. This was validated by using CD-HIT [60] to produce sets of non-redundant representative sequences (query coverage ≥80%, 0.8 sequence identity cut-off). The putative protein coded for each cluster was identified through performing BLASTP on a representative amino acid sequence from each cluster. Comparison of the annotated genomes were also carried out in MicroScope server [61].

Further, the coding sequences were processed for functional annotation using the bi-directional best-hit (BBH) assignment method on KEGG Automatic Annotation Server (KAAS) [62]. This annotation was then used for biological family construction using protein family prediction on MinPath [63]. The top 50 subsystems were selected based on normalized values obtained by dividing with the lowest value for the genes in the respective pathways. Finally, the nine *Sphingobium* strains and enriched pathways were clustered heirarchially using Pearson correlation with 0.8% minimum abundance and a heat map was constructed in MeV4.9.0 [64]. Genomic Islands (GIs) were analysed using the IslandViewer software tool (http://www.pathogenomics.sfu.ca/islandviewer) [65]. The CRISPR Finder online server was used to identify CRISPR elements in the draft genomes [66], which were further analyzed to trace their sources.

### Phylogenetic analysis of *Sphingobium*spp

The phylogenetic analyses were performed using four different methods.

i) **16S rRNA gene sequences:** which were retrieved using BLASTN (E-value = 10^-5^) [59]. The 16S rRNA sequences were aligned using CLUSTALX [67] and subsequently a phylogenetic tree was constructed using the TreeconW software package version 1.3b [68] with the Jukes & Cantor model (1969) [69] and Neighbor Joining algorithm (bootstrap value = 1000).
ii) **Single Copy Gene Sequences:** The amino acid sequences of 28 universally present single copy genes (*dnaA*, *frr*, *gyrB*, *infB*, *mnmA*, *nusA*, *pheS*, *rplB*, *rplC*, *rplM*, *rplS*, *rpoA*, *rpsB*, *rpsC*, *rpsH*, *rpsI*, *rpsJ*, *rpsS*, *trmD*, *tef*, *ychF*, *alaS*, *rplE*, *uvrC*, *lepA*, *rplI*, *rplP* and *rplD*) were retrieved and concatenated. Further, they were aligned using CLUSTALX (as described above) and their phylogeny was constructed using TreeconW software package version 1.3b with the Poisson correction model and Neighbor Joining algorithm (bootstrap value = 1000).
iii) **Tetranucleotide Correlation:** Whole-genome based tetranucleotide correlation was performed using TETRA software [70], based on which a Pearson correlation matrix was constructed. This was followed by hierarchial clustering on the resultant matrix using MeV4.9.0 [39] and finally a dendrogram was constructed in MEGA4 [71].
iv) **Average Nucleotide Identity (ANI):** This method includes all possible pairwise comparisons between these genomes as described by Konstantinidis and Tiedje (2005) [18]. Pearson correlation matrices were constructed from these ANI values, which were then used to perform hierarchical clustering followed by a dendrogram construction as described above.

### Identification of genes under diversifying natural selection

Orthologs, genes involved in the degradation of aromatic compounds including HCH, and genes for transposable elements were analyzed for positive selection by extracting their sequences and performing codon by codon alignment on CLUSTALX [67]. These dN/dS and dS values, calculated for each gene pair using Hyphy 2.1.2 [72], were plotted to show the time-independent evolution of the genes.

### Arrangement and diversification of the *lin*catabolic system

The Artemis Comparison Tool (Web-ACT) [73] was used to compare the arrangement of the *lin* genes and proximal genetic mobility elements such as IS*6100* with reference to *lin* arrangement in *S. japonicum* UT26S. After constructing a database of the contigs for each strain, genes for HCH degradation were extracted using BLASTN [59], and the percent identity to the respective gene in the archetypal strain UT26S was used as a measure of genetic divergence. This divergence was plotted in addition to copy number values for each gene in each strain using the R package ggplot2 [74]. Principle component analysis (PCA) with the copy number and divergence data was then done with the R package FactoMineR [75], with HCH degradation plotted as a supplementary discrete variable (0- complete non degrader, 1- partial degrader, 2- full degrader), followed by a plot construction with ggplot2. Recruitment plots of the raw reads of the six *Sphingobium* spp. mapped against the sequence of all the plasmids of *Sphingomonas* sp MM-1 (extracted from NCBI) were created using MUMmer 3.23 [76].

## Availability of supporting data

The supporting data has been deposited in Dryad (http://datadryad.org/) with doi:10.5061/dryad.g7t27.

## Electronic supplementary material

Additional file 1: Table S1: Genes cluster identified for the degradation of aromatic hydrocarbons. **Table S2.** *lin* genes copy number within the *Sphingobium* genomes. (DOCX 45 KB)
