# Supplementary material for: Comparative genomic analysis of nine Sphingobium strains: insights into their evolution and hexachlorocyclohexane (HCH) degradation pathways
Source: BMC Genomics. 2014 Nov 23;15(1):1014. doi: 10.1186/1471-2164-15-1014 (PMC4289293; doi:10.1186/1471-2164-15-1014)
Supplement: Supplementary file 1 — Additional file 1: Table S1: Genes cluster identified for the degradation of aromatic hydrocarbons. Table S2. lin genes copy number within the Sphingobium genomes. (DOCX 45 KB) [file 12864_2014_6830_MOESM1_ESM.docx]

**Table S1: Genes cluster identified for the degradation of aromatic hydrocarbons**

(+ present; - absent)

**Table S2: *lin* genes copy number within the *Sphingobium* genomes**

|  | ***lin*A** | ***lin*B** | ***lin*C** | ***lin*DER** | ***lin*F** | ***lin*GHIJ** | ***lin*KLMN** |
| --- | --- | --- | --- | --- | --- | --- | --- |
| **IP26** | **1** | **1** | **1** | **1** | **1** | **1** | **1** |
| **HDIPO4** | **1** | **2** | **1** | **2** | **2** | **1** | **1** |
| **RL3** | **1** | **1** | **-** | **3** | **1** | **-** | **1** |
| **P25** | **1** | **-** | **-** | **-** | **1** | **-** | **1** |
| **DS20** | **-** | **-** | **-** | **-** | **-** | **-** | **1** |
| **LL03** | **1** | **-** | **-** | **1** | **1** | **1** | **1** |
| **B90A** | **2** | **1** | **1** | **1** | **1** | **2** | **1** |
| **UT26S** | **1** | **1** | **1** | **1** | **1** | **1** | **1** |
| **SYK6** | **-** | **-** | **-** | **-** | **-** | **-** | **-** |
